# Curvature-driven bubbles or droplets on the spiral surface

**DOI:** 10.1038/srep37888

**Published:** 2016-11-25

**Authors:** Shanpeng Li, Jianlin Liu, Jian Hou

**Affiliations:** 1 College of Pipeline and Civil Engineering, China University of Petroleum (East China), Qingdao 266580, China; 2 College of Petroleum Engineering, China University of Petroleum (East China), Qingdao 266580, China

## Abstract

Directional motion of droplets or bubbles can often be observed in nature and our daily life, and this phenomenon holds great potential in many engineering areas. The study shows that droplets or bubbles can be driven to migrate perpetually on some special substrates, such as the Archimedean spiral, the logarithmic spiral and a cantilever sheet in large deflection. It is found that a bubble approaches or deviates from the position with highest curvature of the substrate, when it is on the concave or convex side. This fact is helpful to explain the repelling water capability of *Nepenthes alata*. Based on the force and energy analysis, the mechanism of the bubble migration is well addressed. These findings pave a new way to accurately manipulate droplet or bubble movement, which bring inspirations to the design of microfluidic and water harvesting devices, as well as oil displacement and ore filtration.

Directional motion of droplets can often be observed in nature and our daily life, where many plants and animals can realize some biological functions based on this fact. For example, high trees can transport water from the soil to their leaves via small capillary tubes in the trunk. The cactus in dry deserts can efficiently collect fogs from the air by using the special microstrucstures in the conical spines^1^,^2^. Moreover, different from lotus, the leaves of rice have anisotropic structures, i.e. the parallel channels, which can repel water outside in one certain direction. Similar examples can also be noticed in the animal world. For instance, a beetle in the Namib Desert is able to collect dews as it has special surface microstructures on the carapace^3^. Besides this, it is reported that the spider web possesses the strong capability to gather water from air by using the reorganized structure of the puffs, and this skill brings inspirations of designing manmade devices to catch water^4^. More surprisingly, the Texas horned lizard can even suck water from mud by the thin channels spreading from its feet to mouth^5^; and a kind of shorebird can make its beak in a tweezering motion to realize the surface tension-induced transportation of prey^6^.

How to accurately control the directional motion of droplets or bubbles is crucial to applications of micro-fluidic devices in such areas as bioassays, microreactors, and chemical or biological sensing^7^, as well as the collection of oils and displacement of petroleum. Several methods have been applied to achieve the directional movement of droplets by modulating the surface tension of the environmental liquid^8^,^9^, the temperature gradient^10^,^11^,^12^, the wettability variation of the substrate surface^13^,^14^,^15^,^16^,^17^,^18^,^19^,^20^, and the stiffness of the substrate^21^. In practice, the surface tension difference of liquid can even be used to actuate the motion of a small plastic plate^22^, or a microboat for potentially transporting desired targets^23^. However, even without these external factors, the substrate with special geometric shapes can also drive the droplets to move directionally^24^,^25^,^26^,^27^,^28^,^29^,^30^,^31^. When a droplet is deposited on a conical fiber, inside a conical tube or between two nonparallel plates, it can spontaneously move due to the pressure gradient difference. In addition, droplets or bubbles placed in the vicinity of a liquid meniscus can also be propelled or rejected naturally^32^,^33^.

It can be affirmed that the main reason of the geometry-driven droplet or bubble motion is attributed to the nonsymmetrical shape of the substrate. Consequently, it is natural to concentrate on the spiral-shaped substrate, whose curvature can always change at different positions. As is well known, there are all kinds of spirals existing in nature, such as the nebula, sunflower seed array, grapevine, snail shell and even DNA. The mystique of spiral may be that it symbolizes “growth”, as it has the property “*Eadem mutata resurgo*”^34^. Therefore, we investigate how a droplet or bubble migrates when placed on a spiral-like substrate, including an Archimedean spiral, a logarithmic spiral and a cantilever sheet in large deflection. It is found that the droplet or bubble moves directionally on these kinds of surfaces, and we address the mechanism from the viewpoint of force and energy analysis.

## Results

### Migration of a droplet or bubble on an Archimedean spiral

**Figure 1:**
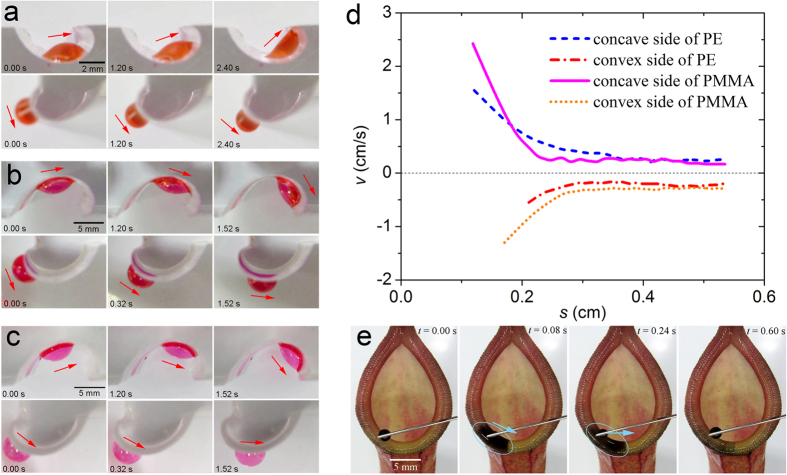
Migration of a droplet or bubble on an spiral. (**a**) Snapshots of a droplet moving on the concave side (upper row) or convex side (lower row) of the Archimedean spiral made of PMMA. (**b**) Snapshots of a bubble moving on the concave side (upper row) or convex side (lower row) of the Archimedean spiral made of PMMA. (**c)** Snapshots of a bubble moving on the concave side (upper row) or convex side (lower row) of the Archimedean spiral made of PE. (**d**) The velocity of a bubble moving on the concave or convex side of an Archimedean spiral made of PMMA or PE. (**e**) Snapshots of a droplet deposited on the concave side of the pitcher rim of *Nepenthes alata*. The droplet spreads and moves towards the position of the logarithmic spiral with highest curvature quickly, with a decreasing volume (marked with blue arrows). In order to remove the effect of the droplet’s gravity, the spiral rim is set horizontal.

An Archimedean spiral is placed parallel to the horizontal surface, which is made of hydrophilic polymethylmethacrylate (PMMA), as shown in Fig. 1a–c (top view). The mathematical function of the spiral reads


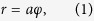


where *r* is the polar radius, *φ* is the polar angle, and the parameter *a* = 1/360 cm/° (Supplementary, Fig. S1). A small droplet with the volume of 0.01 mL is released on the concave side of the spiral. It is found that the droplet spontaneously moves towards the spiral center, as shown the snapshots in the upper row of Fig. 1a. Then the droplet is put on the convex side of the same spiral, and it deviates from the center, shown as the snapshots in the lower row of Fig. 1a. To get a clear picture, the droplet is dyed into red by the Eosin Y water solution (from the Jining Hongwei Chemical Company). However, due to the gravitational effect, the orbit of the droplet is not a perfect spiral.

As a consequence, we use a bubble to replace the droplet, in order to remove the influence of gravity, as its thickness is much smaller than the radius. First, a spot of dishwashing detergent is added into the bubble to make it more stable, and the bubble is also dyed into red by the Eosin Y water solution. As is pointed out that the Young’s contact angle of liquid on a solid surface is immeasurable^35^,^36^,^37^, thus we measure the values of advancing angle *θ*_A_ and receding angle *θ*_R_ of a bubble on PMMA. Although there are so many methods to measure them, for the purpose of convenience, the method of inflating and deflating of a bubble^38^,^39^,^40^ is used, and the two angles are given as *θ*_A_ = 89 ± 3° and *θ*_R_ = 85 ± 2°. The surface tension of the water including dishwashing detergent is measured as 28 mN/m by using the pendant droplet method. The bubble with the volume of 0.05 mL is gently deposited on the surface of an Archimedean spiral, whose spiral distance *a* = 1/180 cm/°, as shown in Fig. 1b. In this case, the trajectory of the bubble is a perfect spiral, which is parallel to the horizontal surface, as its gravity can be ignored. It is observed that, when the bubble is on the concave side of the spiral, it initially approaches the spiral center slowly, and then speeds up. Oppositely, when the bubble is put on the convex side of the spiral, it departs from the center quickly, and finally attains to a constant speed due to the friction of the surface. Next, we place the bubble on a spiral made of polyethylene (PE), whose advancing angle *θ*_A_ = 95 ± 4° and receding angle *θ*_R_ = 88 ± 2°, i.e. the wetting property of the substrate is close to weak hydrophobicity. In this situation, the bubble also moves to the spiral center when it is on the concave side, and deviates from the center when on the convex side, as demonstrated in Fig. 1c. It should be mentioned that if there is no friction, the bubble can move perpetually on the spiral, but this is impossible in reality. In the experiment, the distances of a bubble can travel on PE and PMMA spirals are very close, which both arrange from 0.43 to 0.56 cm.

The velocities of a bubble on various spiral surfaces are plotted in Fig. 1d, where the arc length *s* along the spiral measured from the center is introduced to locate the bubble. For clarity, we define the sign of the velocity as follows: when the direction of the bubble is towards the spiral center, it is positive; and vice versa it is negative. From the figure we can see that the bubble’s speed does not change much when it is far away from the center, where the curvature of the spiral becomes smaller; and the bubble moves quickly when it approaches the center, where the curvature is bigger. Obviously, the velocity-distance curve can be fitted as *v* ∝ *s*^*a*^, when the bubble is close to the spiral center. For a bubble on the concave and convex side of PE, the exponent *α* is approximated as −1.480 and −1.242, respectively; and for a bubble on the concave and convex side of PMMA, the exponent is estimated as −2.393 and −1.854, respectively. It is shown that the speed of a bubble on the concave side is bigger than that on the convex side of the same spiral. Moreover, when the bubble is on the same side of the spiral, its speed is bigger when it is on PMMA (2.45 cm/s on the concave side and 1.30 cm/s on the convex side) than that on PE (1.55 cm/s on the concave side and 0.55 cm/s on the convex side), indicating that wettability of the substrate can significantly affect the bubbles’ velocity.

### Migration of a droplet on a logarithmic spiral

In fact, a lot of spirals in nature take the logarithmic format, which is also named as “growth spiral”. For example, the pitcher rim of *Nepenthes alata* can be roughly viewed as a logarithmic spiral *r* = 0.319*e*^0.0181*φ*^, as shown in Fig. 1e. This structure is helpful for the *Nepenthes alata* to avoid water flowing into the pitcher, as too much water can dilute the digestive juice inside it. It has been reported that the droplet can be pushed from the inside to outside of the pitcher rim, with the aid of hierarchical structure of *Nepenthes alata*^41^. However, when the amount of small droplets accumulates to a certain value, it is not easy to repel them outside by the hierarchical structure alone. It can be seen that the spiral characteristic of the rim may take effect to remove the water.

We deposit a black droplet dyed by Direct Black 19 (from the Hebei Chang Lu Chemical Dyes Company) on the rim of *Nepenthes alata*, as shown in Fig. 1e (top view). The rim, i.e. the logarithmic spiral, is made horizontal to avoid the gravitational effect of the droplet. It is observed that the droplet flows along the concave side of the spiral rim to the position with highest curvature, i.e. the bottom point in Fig. 1e. In this process, a portion of water has already been repelled outside of the rim, and the volume of the droplet decreases when it moves, indicating that the hierarchical structure functions. It manifests that the combination of the spiral-induced propulsion and hierarchical structure of *Nepenthes alata* contributes to propel water quickly and efficiently. This capability may be vital to the survival of *Nepenthes alata*, as it always grows in a humid environment, which can normally produce many droplets.

### Migration of a bubble on a sheet with large deflection

**Figure 2:**
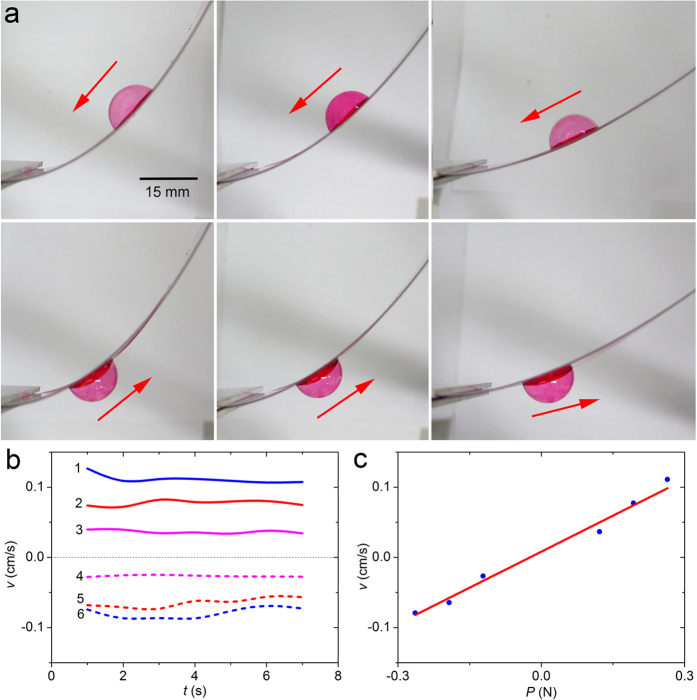
Migration of a bubble on the concave or convex side of a cantilever sheet with large deflection. (**a**) Snapshots of the migration of a bubble on the concave (upper row) or convex (lower row) side of a cantilever sheet. The three subfigures in both of the upper and lower rows correspond to the case that the external force *P* = 0.26, 0.19 and 0.12 N, respectively. (**b**) Velocity of the bubble on the concave or convex side of the spiral made of PMMA or PE. The three groups, i.e., lines 1 & 6, lines 2 & 5 and lines 3 & 4 correspond to the case that the external force *P* = 0.26, 0.19 and 0.12 N, respectively. Solid lines 1, 2, 3 and dash lines 4, 5, 6 correspond to the concave side and convex side, respectively. (**c**) Linear relationship between the velocity of the bubble and external load.

Although the bubble can be driven on spirals, it is not convenient to fabricate them in practice. In order to realize the real-time control of bubbles, we use a cantilever sheet in large deflection as the substrate, whose curvature can also change along the axis. The material of the sheet is Polyethylene Terephthalate (PET), with the advancing angle *θ*_A_ = 89 ± 3° and receding angle *θ*_R_ = 86 ± 3°, length *L* = 70.8 mm, width *b* = 33.05 mm, and thickness *t* = 0.39 mm. The left end of the cantilever is fixed, and there is a concentrated force at the free end, normal to its original axis. The axis of the sheet is parallel to the horizontal plane, and the external force causes the large deflection of the cantilever. As shown in Fig. 2a (top view), a bubble is put on the surface of the cantilever. The snapshots show that the bubble moves to the left end of the sheet when it is on the concave side (upper row), and to the right when on the convex side (lower row). Due to the existence of friction, the bubble finally stops when it travels 0.52–1.18 cm on the convex surface of the beam.

The velocities of the bubble under the action of the force *P* = 0.12, 0.19 and 0.26 N are respectively displayed in Fig. 2b. It shows that, when the force is given, the velocity of the bubble does not change much, which is different from the case on the spiral substrate. This may be owing to that the curvature gradient of the cantilever sheet does not alter violently. It is found that the bigger force causes bigger deformation of the sheet, and then the bubble has a higher speed. It is also noticed that the speed of a bubble on the concave side is bigger than that on the convex side. The dependence relationship between the external force and the bubble velocity is displayed in Fig. 2c, and a linear function can be fitted as 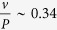. It manifests that the way of mechanics can be efficiently used to control the direction and speed of bubbles.

## Discussion

The first impression on the bubble or droplet moving on a spiral surface is that the process is governed by the Newton’s second law *F*_*d*_ − *F*_*f*_ = *ma*, where *F*_*d*_ is the driving force*, F*_*f*_ the frictional force, *m* the mass of the bubble or droplet, and *a* its acceleration. If the driving force *F*_*d*_ is bigger than the frictional force *F*_*f*_, the bubble or droplet can be propelled forward. As the configuration of the bubble or droplet always changes when it is moving on the spiral-shaped substrate, the driving force and frictional force are different when it is at different positions. When the bubble or the droplet is near the spiral center, the driving force is much bigger than the frictional force; however when it is far away from the center, there is no significant difference between the magnitudes of the two forces, and the velocity approaches a constant. Due to the wetting hysteresis, it is also found that the droplet’s volume must reach a certain value to ensure that its two sides can “feel” the curvature gradient of the substrate, which is big enough to drive the bubble’s migration. For a bubble on the Archimedean spiral made of PMMA and PE with *a* = 1/360 cm/°, the minimum volumes to move are 2.5 and 3 μl, respectively; and if *a* = 1/180 cm/°, the minimum volumes of the bubble to move are 7 and 9 μl, respectively. This is because the curvature gradient of the latter spiral is smaller, and therefore a bigger bubble to move is needed to “feel” this curvature gradient. Similarly, if the bending degree of a cantilever is not significant, it is also not easy for a bubble to move on it. For example, if the bubble’s volume is 0.5 ml, only when the minimum slope angle of the beam’s free end attains around 20°, can the bubble start to move.

**Figure 3:**
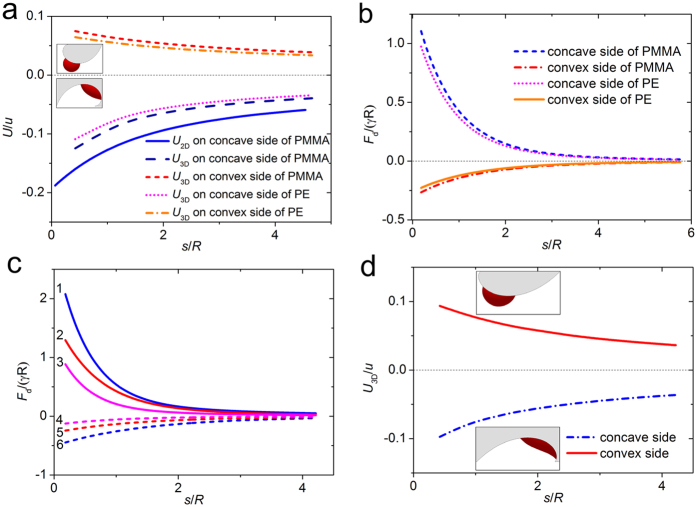
Free energy of a bubble on the spiral. (**a**) The dimensionless energy versus dimensionless distance of bubble. The solid line is the analytical free energy curve in 2D for a bubble on the concave side of PMMA Archimedes spiral, and the other lines are the dimensionless free energy curves in all conditions on the Archimedean spiral in 3D. The Young’s contact angle of the bubble on PMMA and PE are selected as *θ*_Y_ = 87.8° and *θ*_Y_ = 93.1°, respectively. Insets are the simulated pictures of a bubble on the concave side and convex side. (**b**) The dimensionless driving force versus the dimensionless distance of the bubble from the spiral center. (**c**) The dimensionless driving force versus the dimensionless distance for different contact angles. The three groups, i.e., lines 1 & 6, lines 2 & 5, and lines 3 & 4 correspond to the case that *θ*_Y_ = 60°, 90° and 120°, respectively. Solid lines 1, 2, 3, and dashed lines 4, 5 6 correspond to the concave side and convex side, respectively. (**d**) The free energy curves of the bubble on a logarithmic spiral with *θ*_Y_ = 60° in 3D. Insets are the simulated pictures of a bubble on the concave and convex side of the spiral.

To get a clearer landscape on the motion of a bubble, it is necessary to perform the energy analysis of the whole system. Without loss of generality, we consider the configuration of a bubble on an Archimedean spiral in two-dimensional (2D) case. Considering the two liquid/vapor interfaces of the bubble, the free energy of the system is expressed as:


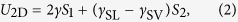


where *S*_1_ and *S*_2_ represent the areas of the vapor/liquid and solid/liquid interface, *γ*_SV_, *γ*_SL_ and *γ* are the interfacial tensions of solid/vapor, solid/liquid and liquid/vapor, respectively. In the theoretical model, the roughness of the substrate surface is omitted, and it is assumed that the Young’s contact angle *θ*_Y_ = *θ*_A_ = *θ*_R_. In consideration of the geometric relations (the expression of the apparent angle in Fig. S2 is consistent with the previous result^42^), the value of *U*_2D_ can be derived (Supplementary). To verify the theoretical result, the free software *Surface Evolver* is used to simulate this process in three-dimension (3D), and the free energy of the system *U*_3D_ can be obtained^43^. The dependence relationship between the free energy and the arc length *s* can be plotted in Fig. 3a. The free energy *U* is normalized by *u*, which equals 4*γ*π*R* or 8*γ*π*R*^2^ for the 2D or 3D case, respectively, and the arc length is rescaled by *R*, which is the radius of the spherical bubble before deposited on the substrate. The result shows that the 2D analysis is in excellent agreement with the 3D result. When the bubble on the concave side of the spiral approaches its center, the free energy becomes smaller; and vice versa, when on the convex side and approaching the spiral center, the free energy becomes bigger. Obviously, the bubble always tends to the position with lowest free energy, according to the principle of least action, and this analysis is in accordance with the experimental observations.

The driving force of the bubble can be deduced as the gradient of the free energy with respect to the arc length, i.e. 
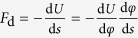
, where 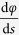 is the curvature of the substrate. For the Archimedean and logarithmic spiral, 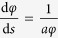 or 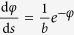. Then the curve of the driving force versus the arc length *s* is presented in Fig. 3b. It shows that when the bubble is near the spiral center, the curvature is bigger, and the driving force becomes bigger, thus the velocity of the bubble is higher, and this phenomenon agrees well with the experimental observation. The impact of the Young’s contact angle on the driving force is shown in Fig. 3c, where *θ*_Y_ is selected as 60°, 90°, 120°, respectively. The curve indicates that stronger hydrophilicity of the substrate leads to a bigger driving force, and this conclusion is consistent with the experiment. Similarly, for a bubble on the concave or convex side of a logarithmic spiral, the free energy of the system is demonstrated in Fig. 3d, simulated by *Surface Evolver*. It manifests that for a bubble coming close to the spiral center, the free energy becomes bigger when it is on the concave side, and becomes smaller when on the convex side.

**Figure 4:**
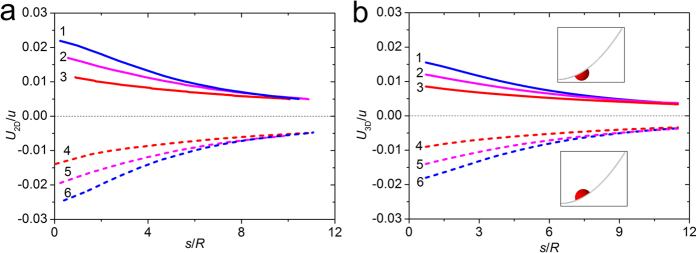
Free energy of a bubble on a cantilever sheet in large deflection. (**a**) The dimensionless free energy curves of bubble on the sheet with different deflections in 2D. (**b**) The dimensionless free energy curves of a bubble on the sheet with different deflections in 3D. The Young’s contact is selected as *θ*_Y_ = 88.4°. Insets are the simulated pictures of the bubble in the cantilever sheet. Both in (**a**) and (**b**), the three groups, i.e., lines 1 & 6, lines 2 & 5 and lines 3 & 4 correspond to the case that the external force *P* = 0.26, 0.19 and 0.12 N, respectively. Solid lines 1, 2, 3 and dashed lines 4, 5, 6 correspond to the convex and concave side, respectively.

For a bubble on a cantilever sheet, the external force *P* induces the deformation of the sheet as follows^44^:


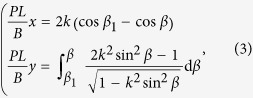


where 1 + sin *ϕ* = *2k*^2^sin^2^*β* = (1 + sin *ϕ*_0_)sin^2^*β*, 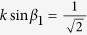, and *B* is the bending stiffness. Similar to the bubble on an Archimedean spiral, the free energy of the system in 2D can be derived, and the numerical result via *Surface Evolver* is also given as a comparison, then the curves are demonstrated in Fig. 4. It also shows when the bubble approaches the clamped end, the system is of the lowest free energy when it is on the concave side, and of highest energy when on the convex side of the sheet. Therefore, the bubble selects its proper route to reach the position of lowest energy, which is in consistent with the experimental phenomenon. The slope of the curve represents the magnitude of the driving force, which indicates that when the bubble is close to the fixed end, the driving force is bigger. However, compared with the spiral, the curvature alteration of the cantilever sheet looks gentle, so the observed velocity of the bubble is nearly a constant.

In summary, the migration of a bubble or droplet moving on a spiral-shaped substrate is comprehensively studied. It is found that for a bubble placed on an Archimedean spiral, a logarithmic spiral or a cantilever sheet in large deflection, it migrates towards the spiral center when on the concave side, and deviates from the center when on the convex side. The driving force is correlated with the curvature of the substrate, manifesting that when the bubble comes close to the position with highest curvature, the velocity becomes bigger. The wettability of the substrate does not affect the migration direction of the bubble, but can alter the speed of the bubble. All of these experimental results can be well illustrated by the energy analysis based on theoretical model in 2D and software simulation in 3D. Moreover, the cantilever-bubble system provides a flexible, real-time and non-contact control of bubble and droplet movement.

## Methods

### Experiments of the bubble formation

The bubble is generated by a medical injector. First, we pull out the syringe plunger to make sure the syringe is filled with air. Then we put the injector needle into the surfactant solution, and when it leaves the solution, there is a liquid film around the needle. In succession, we slowly push the plunger, and the extruded air together with the liquid film form a bubble with a certain volume, according to the volume mark on the injector.

### Experiments of the bubble and droplet moving on spirals

Two Archimedean spirals are fabricated on the basis of a rectangular PMMA plate with the thickness of 19.8 mm, which is formed by laser cutting, with the roughness parameter *R*_*a*_ = 0.2. A thin sheet made of polyethylene (PE) with the thickness of 0.1 mm is then attached on one of the PMMA spirals, which has been made hydrophobic by the vacuum vapor deposition method (with the roughness parameter *R*_*a*_ = 0.05). The two kinds of spirals are placed on the horizontal test bed, whose spiral profiles are parallel to the horizontal surface. The experiments are conducted at room temperature, about 20 °C. The bubble is produced by a medical injector with 1 mL and then is released on the spiral surface, and the snapshots are captured via the camera (Canon SX240HS), whose effective pixel is 12,100,000. A spot of surfactant, i.e. dishwashing detergent is added into water, and their volume ratio is 1:400. The surface tension of liquid is measured using the pendant droplet method adopting the contact angle goniometer (Biolin Scientific Corporation, Thetalit 100). The advancing and receding contact angles of the bubble on PMMA or PE are measured in use of the method of inflating and deflating of a bubble. After the bubble generated by a medical injector is placed on the solid surface, a fine needle in the syringe is used to penetrate into the bubble. As the syringe can control the air inside the bubble, the bubble volume can be increased or decreased correspondingly. The profile and contact angles of the bubble on the solid substrate are captured by the contact angle goniometer (Biolin Scientific Corporation, Thetalit 100).

### Experiment of the bubble moving on the cantilever sheet

The experiment is performed under the room temperature, around 20 °C. The roughness parameter of PET is *R*_*a*_ = 0.025. The value of the force is recorded by the electronic balance (PT-2004/405) with the precision of 0.01 mg. A bubble with the volume 0.5 mL is produced by a medical injector with 1 mL. The motion of the bubble is recorded with a camera (Canon SX240HS).

## Additional Information

**How to cite this article**: Li, S. *et al*. Curvature-driven bubbles or droplets on the spiral surface. *Sci. Rep*. **6**, 37888; doi: 10.1038/srep37888 (2016).

**Publisher’s note:** Springer Nature remains neutral with regard to jurisdictional claims in published maps and institutional affiliations.

## Supplementary Material

Supplementary Information

Supplementary Video S1

Supplementary Video S2
