# Supplementary material for: Curvature-driven bubbles or droplets on the spiral surface
Source: Sci Rep. 2016 Nov 25;6:37888. doi: 10.1038/srep37888 (PMC5122859; doi:10.1038/srep37888)
Supplement: Supplementary Information [file srep37888-s1.doc]

**Curvature-driven bubbles or droplets on the spiral surface**

Shanpeng Li[[1]](#footnote-2), Jianlin Liu^1^ & Jian Hou^2^

**Supplementary materials**

**Formulation of the spiral.** Refer to a Cartesian coordinate system *O*–*xy*, as shown in Fig. S1. A spiral, whether an Archimedean or logarithmic spiral, can be determined in the polar system $O-r\theta$, where *r* is the polar radius of each point on the spiral, and $\theta$ is the polar angle. For the Archimedean spiral, the mathematical expression is

| $r=a\theta$, |  |
| --- | --- |

and for the logarithmic spiral, it is

| $r=be^{\theta}$, |  |
| --- | --- |

where the parameter *a* is the spiral distance, and *b* is also a constant.


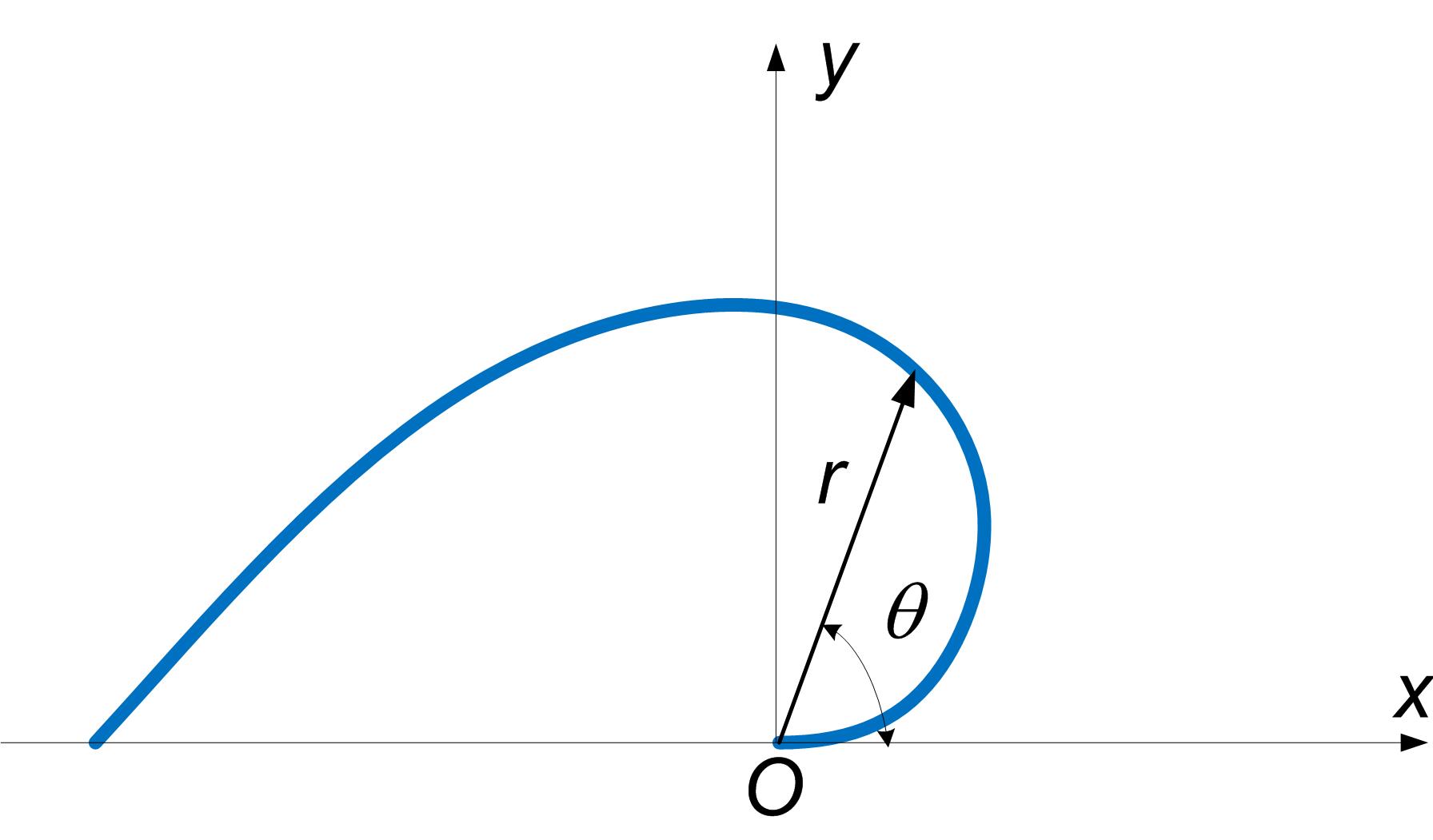


**Figure S1 Schematic of a spiral.**

**Energy analysis of a bubble on an Archimedean spiral in 2D.** Refer to a Cartesian coordinate system *O*′–*xy*, as shown in Fig. S2. The origin of this coordinate system is set at the center of the Archimedean spiral $r=a\theta$. The shape of the bubble is assumed as a portion of a circle, with the radius $\rho$, and the contact angle is selected as the Young’s contact angle, as the surface is smooth enough. The slope angle of each point in the spiral is denoted by . At the triple contact line, the bubble satisfies the Young’s equation

| $\gamma_{\text{SV}}-\gamma_{\text{SL}}=2\gamma\cos\theta_{\text{Y}}$, |  |
| --- | --- |

as the bubble has two liquid/vapor interfaces, where $\theta_{\text{Y}}$ is the Young’s contact angle. Thus, the total energy of system can be expressed as

|  |  |
| --- | --- |
| $=2\gamma S_{1}-2\gamma S_{2}\cos\theta_{\text{Y}}$, |

where

| , |  |
| --- | --- |
|  |  |
| $=\frac{a}{2}\left(\theta_{\text{B}}\sqrt{\theta_{\text{B}}^{2}+1}-\theta_{\text{A}}\sqrt{\theta_{\text{A}}^{2}+1}+\ln\frac{\theta_{\text{B}}+\sqrt{\theta_{\text{B}}^{2}+1}}{\theta_{\text{A}}+\sqrt{\theta_{\text{A}}^{2}+1}}\right)$, |

where the subscript of the characteristic angle is marked by the corresponding point.


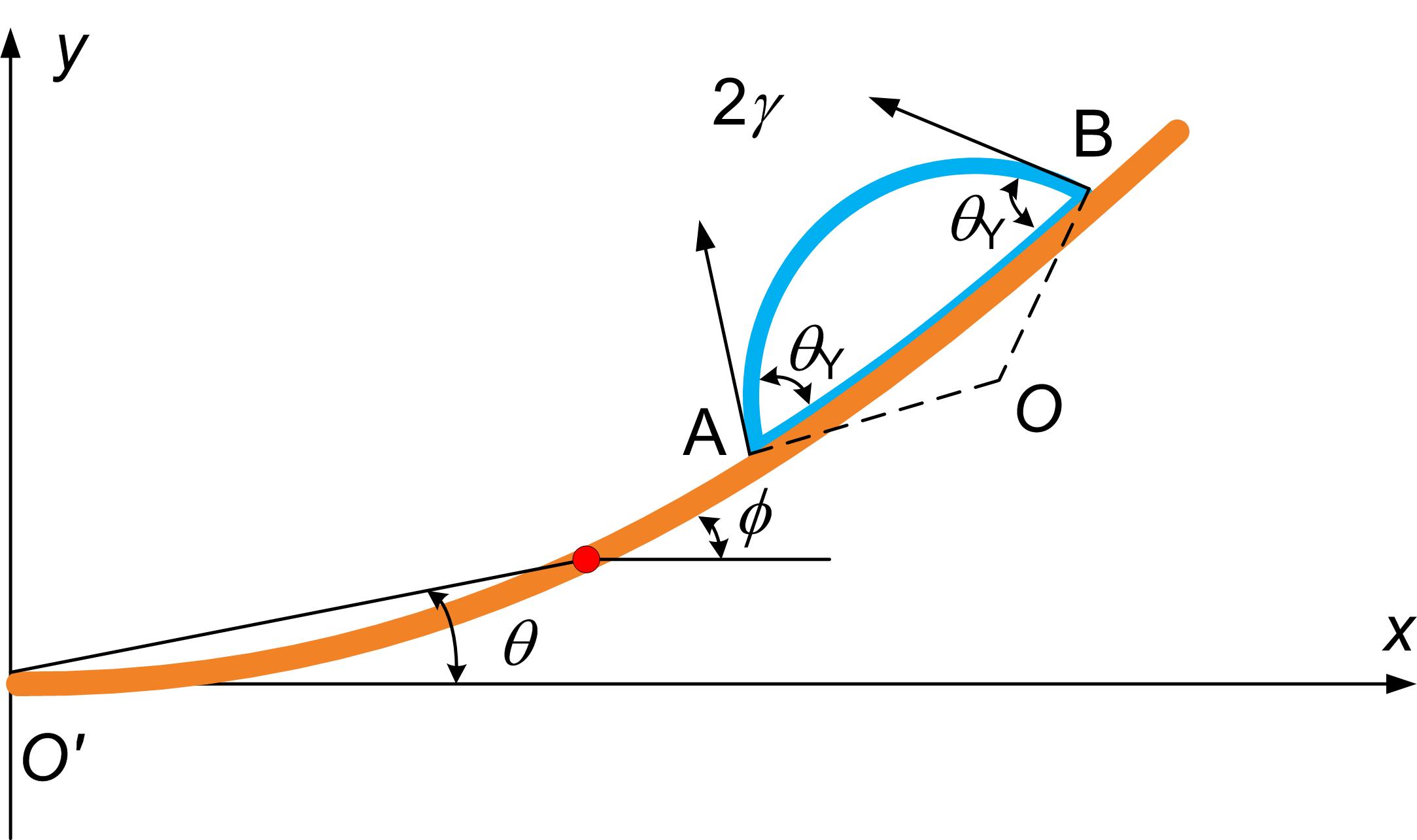


**Figure S2** **Schematic of a bubble on an Archimedean spiral in 2D.**

The related geometric relations are:

| , |  |
| --- | --- |
| , |  |

where the subscript of the coordinate is marked by the corresponding point.

In succession, one has the following relations:

| . |  |
| --- | --- |

Furthermore, the conservation of the bubble volume leads to

| , |  |
| --- | --- |

where *A*_1_ is the volume of the bubble in the current shape, and *A*_o_ is the volume of the bubble before it contacts with the substrate. The expression of *A*_1_ is

|  |  |
| --- | --- |
| $+\frac{1}{2}\left(y_{\text{B}}+y_{\text{A}}\right)\left(x_{\text{B}}-x_{\text{A}}\right)-A_{2}$, |

where *A*_2_ can be derived as:

|  |  |
| --- | --- |
| $=\frac{a^{2}}{12}\left[\left(3\theta_{\text{B}}^{2}\sin2\theta_{\text{B}}-2\theta_{\text{B}}^{3}\right)-\left(3\theta_{\text{A}}^{2}\sin2\theta_{\text{A}}-2\theta_{\text{A}}^{3}\right)\right]$. |

In combination with Eqs. (S4), (S8) and (S10), the free energy of the system can be obtained as a function of the polar angle $\theta$, i. e. . Also we have the relation between the arc length *s* and the polar angle

|  |  |
| --- | --- |
| $=\frac{a}{2}\left[\theta\sqrt{\theta^{2}+1}+\ln\left(\theta+\sqrt{\theta^{2}+1}\right)\right]$. |

Herein, the position of the bubble is designated by point A in Fig. S2. Finally, one can get the free energy curve versus the arc length *s*.

In the same way, when the bubble is on the convex side of the spiral surface, the free energy curve can also be calculated. The only difference is due to the geometric relations, and the derivations have been omitted.

**
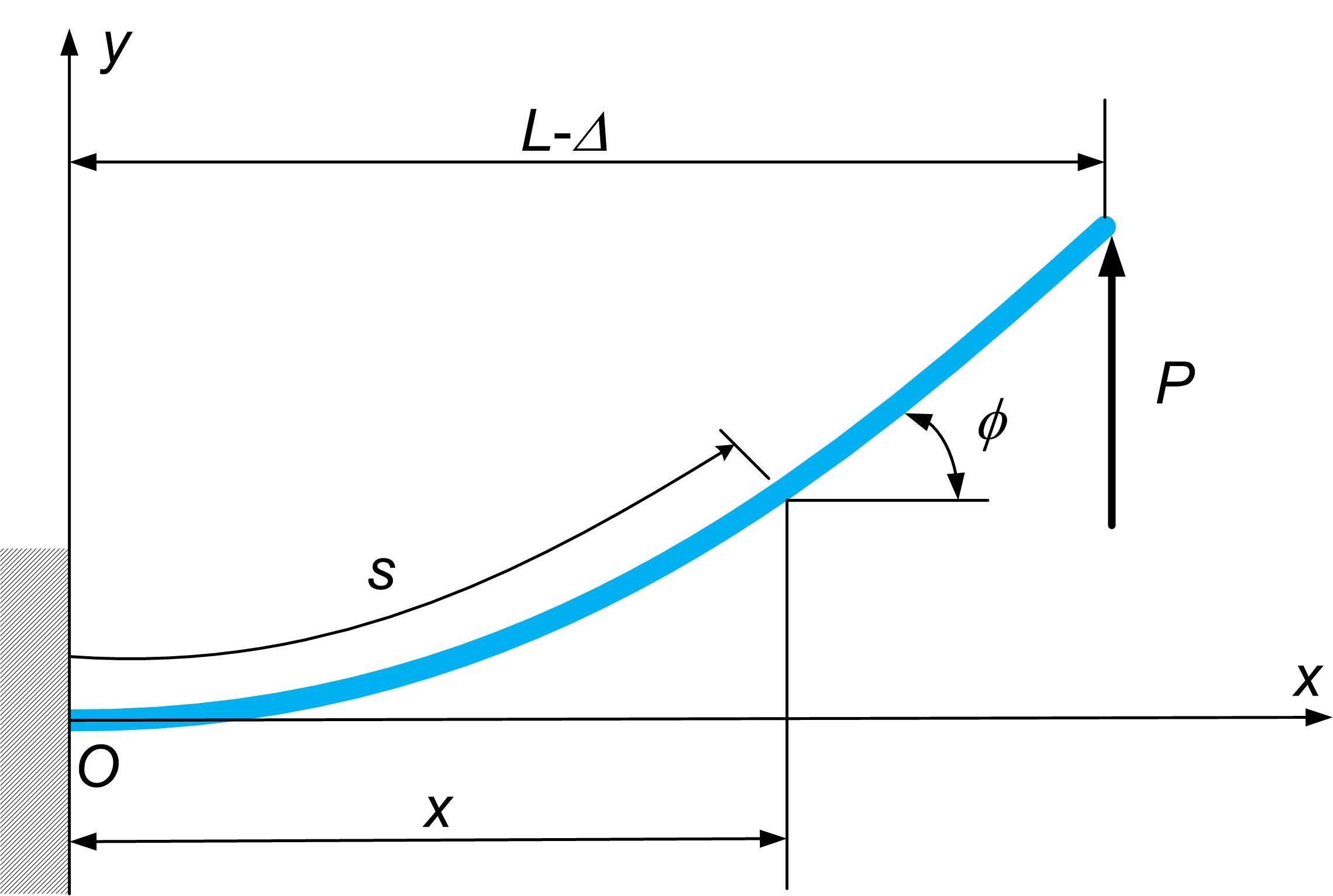
**

**Figure S3 Schematic of a cantilever sheet under the action of a concentrated force in large deflection.**

**Configuration of the cantilever sheet in large deflection.** As shown in Fig. S3, a cantilever sheet demonstrates a large deflection under the action of a concentrated force *P* at the free end. The original length of the sheet is *L*, and the displacement of the free end in the *x* direction is $\Delta$. The parameter is the slope angle of an arbitrary point in the cantilever axis, *B* is the bending stiffness of the sheet, and the arc length *s* is introduced to locate the point. Refer to the Cartesian coordinate system *O–xy*, as shown in Fig. S3. The governing equation of the cantilever sheet is

| , |  |
| --- | --- |

where the dot over a parameter stands for the derivation with respect to *s*. Then one can get

| , |  |
| --- | --- |

where is the slope angle at the free end of the cantilever.

As the sheet is assume inextensible, we have

| . |  |
| --- | --- |

Introducing

| , |  |
| --- | --- |

we can get

| $\left(\begin{matrix} \frac{PL}{B}x=2k\left(\cos\beta_{1}-\cos\beta\right) \\ \frac{PL}{B}y=\int_{\beta_{1}}^{\beta} \frac{2k^{2}\sin^{2}\beta-1}{\sqrt{1-k^{2}\sin^{2}\beta}}\text{d}\beta\end{matrix}\right.$, |  |
| --- | --- |

where $k\sin\beta_{1}=\frac{1}{\sqrt{2}}$.

In calculation, we use a fitting function, i.e. the power function to replace the expression in Eq. (S18). Similar to the former part, i.e. the formulation of the spiral case, the 2D free energy of a bubble on the cantilever sheet can be obtained. Herein, the derivations are omitted.

**Supplementary Video 1.** Video of a droplet deposited on the concave side of the pitcher rim of *Nepenthes alata*.

**Supplementary Video 2.** Video of the migration of a bubble on the surface of a cantilever sheet.

1. College of Pipeline and Civil Engineering, China University of Petroleum (East China), Qingdao 266580, China. ^2^College of Petroleum Engineering, China University of Petroleum (East China), Qingdao 266580, China. Correspondence and requests for materials should be addressed to Jianlin Liu (email: [liujianlin@upc.edu.cn](mailto:liujianlin@upc.edu.cn)) and Jian Hou (email: [houjian@upc.edu.cn](mailto:houjian@upc.edu.cn) ). [↑](#footnote-ref-2)
